# The Clinical and Pathological Characteristics of Malignant Struma Ovarii: An Analysis of 144 Published Patients

**DOI:** 10.3389/fonc.2021.645156

**Published:** 2021-03-05

**Authors:** Yuying Cui, Jinming Yao, Shengnan Wang, Junyu Zhao, Jianjun Dong, Lin Liao

**Affiliations:** ^1^Department of Endocrinology and Metabology, The First Affiliated Hospital of Shandong First Medical University & Shandong Provincial Qianfoshan Hospital, Shandong Key Laboratory of Rheumatic Disease and Translational Medicine, Shandong Institute of Nephrology, Jinan, China; ^2^College of Traditional Chinese Medicine, Shandong University of Traditional Chinese Medicine, Jinan, China; ^3^Department of Endocrinology and Metabology, Shandong University of Traditional Chinese Medicine Affiliated Hospital, Jinan, China; ^4^Department of Endocrinology and Metabology, Qilu Hospital of Shandong University, Cheeloo College of Medicine, Shandong University, Jinan, China

**Keywords:** malignant struma ovarii, thyroid cancer, epidemiology, endocrine tumor, gynecologic oncology

## Abstract

The objective of this study is to summarize the clinical and pathologic characteristics of malignant struma ovarii to facilitate the early diagnosis and treatment of this disease. All 144 patients were females from 27 countries. The mean age of the patients at diagnosis was 42.6 years. Overall, 35.71% of the patients underwent unilateral oophorectomy, 58.57% of the patients underwent bilateral oophorectomy, 5.72% of the patients were not ovariectomized, and 38.57% of the patients received radioactive iodine treatment with an average dose of 158.22 mCI each time. “Impure” types accounted for 70.19% of the cases, while pure types accounted for 29.81% of the cases. Among these cases, papillary thyroid carcinoma accounted for 50.00%, follicular thyroid carcinoma accounted for 26.47%, follicular variant of papillary thyroid carcinoma accounted for 18.63%, papillary and follicular mixed thyroid carcinoma accounted for 2.94%, anaplastic carcinoma accounted for 0.98%, and medullary carcinoma accounted for 0.98%. In total, 21 patients (51.22%) had elevated CA125. More than half of the patients (51.94%) had metastasis outside the ovary. The most common metastatic site was the pelvic cavity. The misdiagnosis rate was 17.27%. Mortality was related to metastasis and the cancer type. Gene mutations were found in the NRAS, KRAS, BRAF, and KIT genes and were similar to those in thyroid carcinoma, but some patients (37.5%) did not exhibit any gene mutations. Regardless of the treatment received, the survival rate is high. Treatment could initially include ovariectomy; however, in cases with metastasis and iodine uptake of the metastatic tumor, thyroidectomy, radioactive iodine therapy, and thyroid hormone inhibiting therapy are indicated.

## Introduction

Malignant struma ovarii (MSO) is a very rare disease. In 1889, follicular thyroid carcinoma tissue was first reported in the ovary (1). MSO was first reported in 1895 (2). Thus far, <200 cases have been reported (3). Malignant struma ovarii is essentially a type of ovarian teratoma that occurs within mature teratomas in 0.1% of the cases. Struma ovarii is a germ cell tumor of the ovary comprised of ≥ 50% thyroid tissue, which should be distinguished from a mature teratoma containing a small component (<50%) of benign thyroid tissue. This tumor mostly appears on one side and appears on both sides in <5% of the cases (4). When the tumor consists entirely of thyroid cancer, it is named pure MSO, and if the tumor consists of thyroid cancer and teratoma components, it is named “impure” MSO (5). Malignant struma ovarii is very rare and accounts for approximately 5% of all struma ovarii.

Roth et al. (6) advocated naming the disease by the location of the thyroid, such as thyroid carcinoma arising from struma ovarii, because the nature of the disease is an ectopic thyroid cancer and a type of teratoma. However, most articles have named this disease MSO.

Research has shown that the gene mutation sites of MSO differed from those of thyroid cancer. The patients' symptoms are very similar to those of ovarian cancer, rendering diagnosis difficult. When patients have obvious symptoms, they often have distant metastases, such as in the liver (7), lungs (8), and bone (9, 10).

Because it is prone to malignant transformation, the management of cases of MSO is still controversial. Most authors (11) have advocated for an aggressive treatment based on local surgery, followed by postoperative adjuvant treatment, including total thyroidectomy (12, 13) and radioactive iodine 131 (I-131) therapy, especially when distant metastases are present.

Both papillary and follicular carcinomas have been reported in struma ovarii, and follicular carcinomas are more common (14, 15), but some researchers believe that the most common pathology is papillary thyroid carcinoma (PTC) (53.18%) (16). The aim of the current study is to gather a sufficiently large cohort of patients to investigate the clinical, pathological, and genetic characteristics of MSO to better understand this rare disease.

## Materials and Methods

### Data Sources and Study Patients

**Figure 1:**
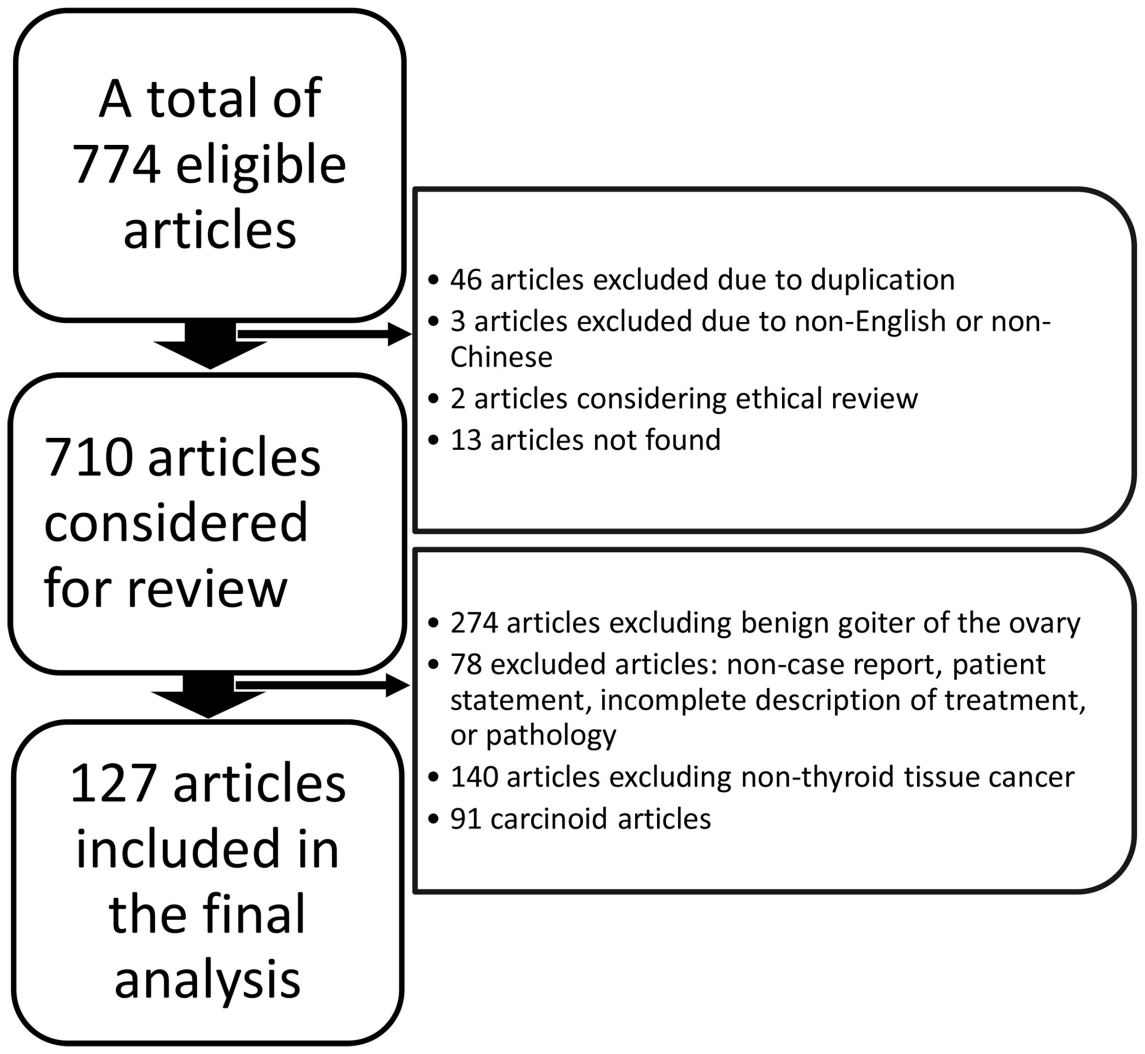
Literature review inclusion process.

We performed a search using the PubMed, ClinicalTrials.gov, Embase, Cochrane Library, and China National Knowledge Infrastructure (CNKI) databases from the date of their inception to 20 March 2020 without language restrictions using the following search terms: “malignant struma ovarii” OR “struma ovarii with malignant transformation” OR “struma ovarii” OR “struma ovarii papillary thyroid carcinoma” OR “struma ovarii follicular thyroid carcinoma.” We included cases of thyroid carcinoma arising in struma ovarii or mature cystic teratoma at the initial ovarian pathological evaluation. All enrolled studies were confirmed based on the following criteria: (i) a clear diagnosis process is presented in the article and (ii) the third edition of the International Classification of Oncology Diseases (ICD-O-3.2) is used as a histological reference for diagnosis. The flow chart (Figure 1) shows the identification of MSO and the reasons for exclusions.

The following clinical and laboratory variables were studied: (i) country; (ii) gender; (iii) age at diagnosis; (iv) type of cancer; (v) whether radioactive iodine treatment was applied and its frequency; (vi) whether the disease was with thyroid disease in the neck; (vii) whether and where the tumors metastasised; (viii) genomic profiling results, including amino acid substitutions and type and position of mutations in the respective genes; (ix) misdiagnoses (Misdiagnosis criteria are obtained from the included literature,); and (x) final results.

### Statistical Analyses

The demographic, clinical, and pathological characteristics of the patients were described utilizing simple summary statistics; the categorical and continuous variables were analyzed utilizing a chi-square analysis and Student's *t*-test.

Statistical analyses were performed using the Statistical Package for the Social Sciences (SPSS) software (version 25.0; SPSS Inc., Chicago, IL); one-sample K-S tests were used to determine whether the data conformed to a normal distribution. All tests were two-sided, and a *p*-value <0.05 was considered statistically significant.

## Results

### Epidemiological Characteristics

**Figure 2:**
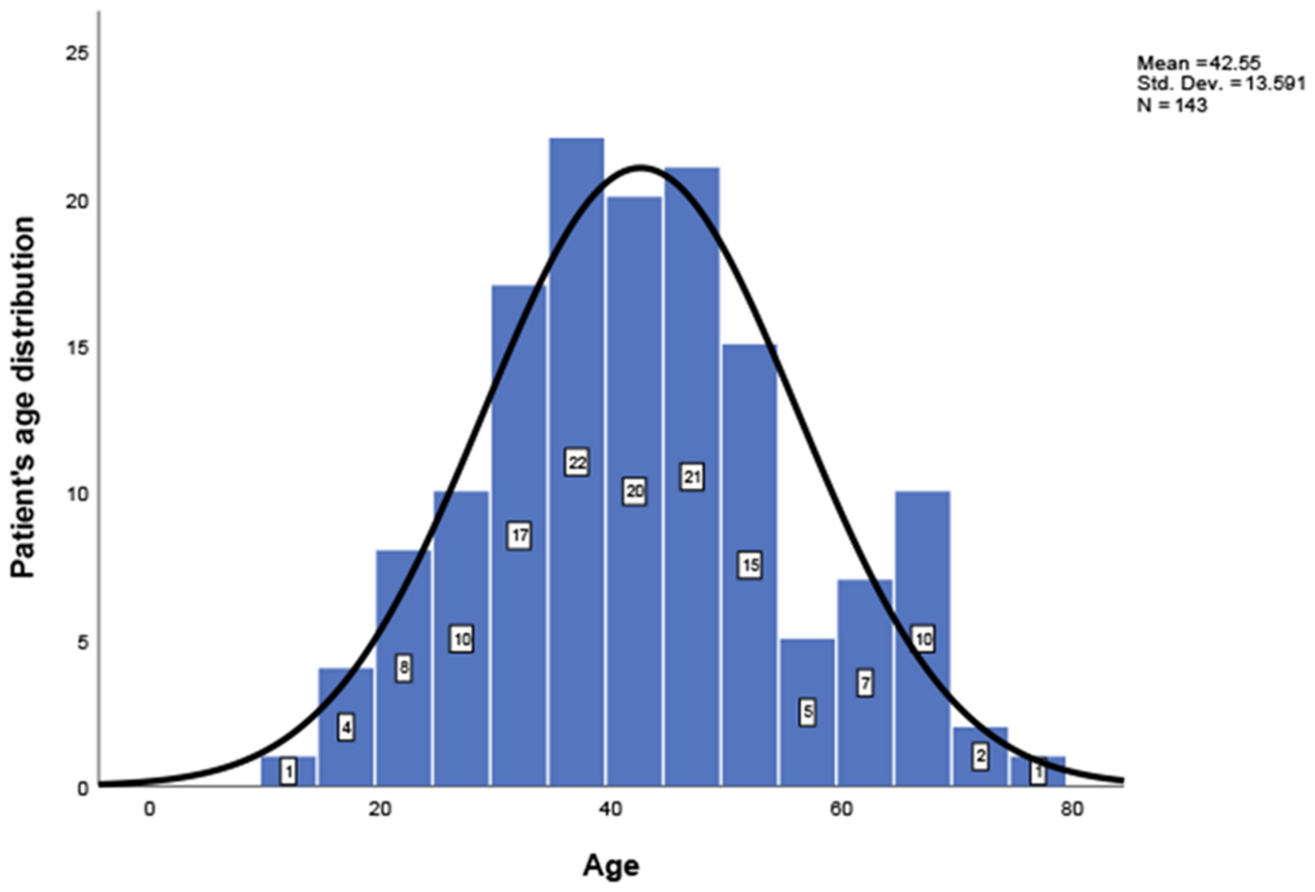
Patients' age distribution; a one-sample Kolmogorov-Smirnov test confirms that the age distribution follows a normal distribution.

**Figure 3:**
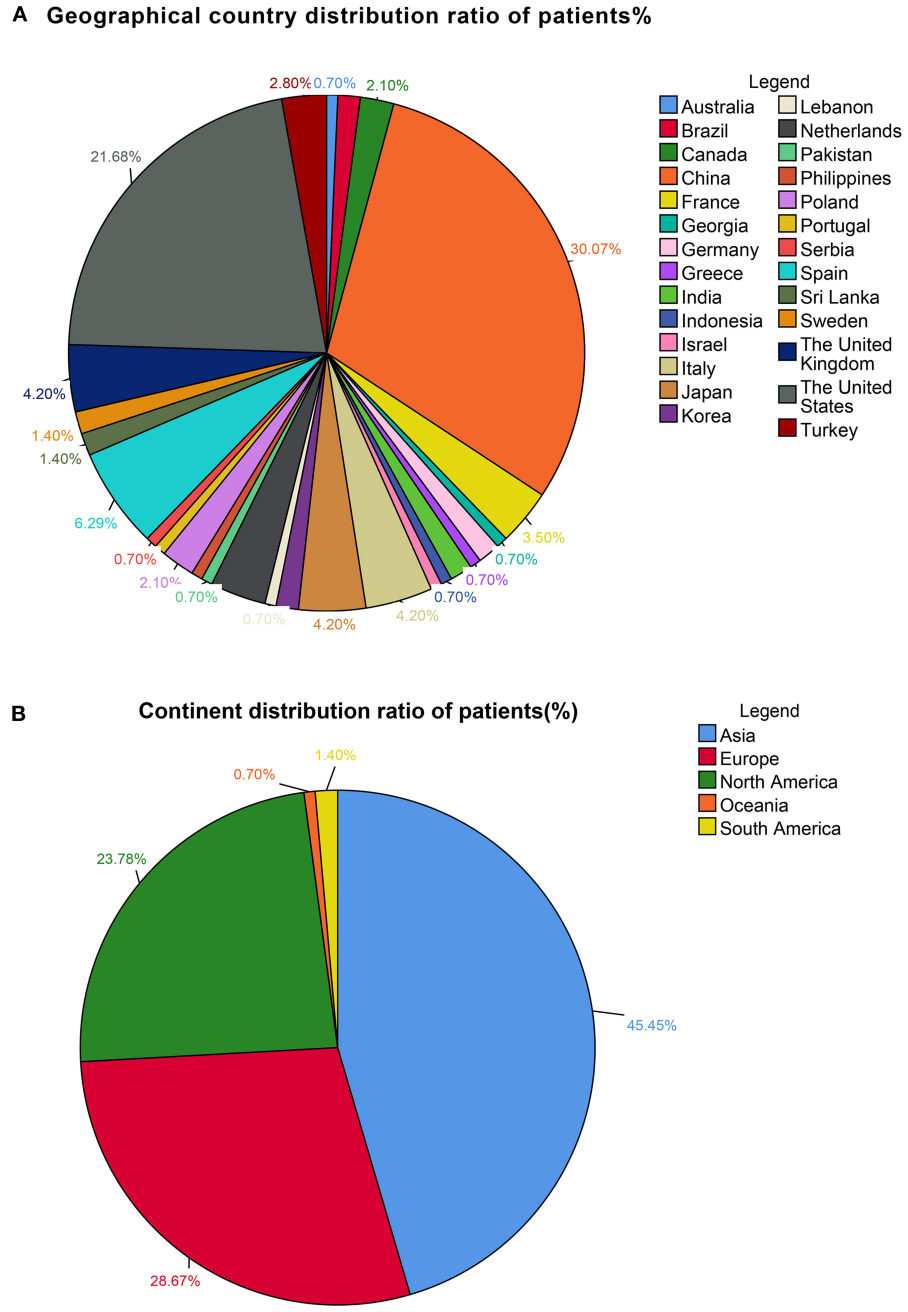
**(A)** Geographical country distribution ratio among the patients (%). **(B)** Continent distribution ratio among the patients (%).

This study included 127 articles (Figure 1) involving 144 female eligible patients from 1970 to 2020. Among the 143 patients with available data regarding age, the average age at diagnosis was 42.55 years (Table 1); the patients' age ranged from 12 to 78 years (Figure 2), and the patients were from 27 countries and 5 continents (Figure 3), including China (42 cases, 29.37%), the United States (32 cases, 22.38%), and Spain (9 cases, 6.29%), which accounted for the top three countries. Asia accounted for the largest proportion of cases (45.45%), and no African case was reported (Figure 3). Among the 75 patients with a clear follow-up time and outcome, the follow-up time ranged from 1 month to 47 years with an average of 77 months (Table 1). Among the 58 patients with complete pregnancy and fertility information, 39 patients had already given birth, and the fertility rate was 67.24%. The highest incidence occurred from the age of 31 to 40 (Figure 2). Patients aged over 60 years only accounted for 13.98% of the cases. We found a special phenomenon, namely, the age at which the patient was diagnosed fit a normal distribution (Figure 2). Except for 18 patients without clear prognostic outcomes and 1 patient lost to follow-up, in total, 125 patients clearly stated the follow-up outcome at the time at which the case was reported; of these patients, 6 died (the mortality rate was 4.8%).

**Table 1:** Summary of the clinical features of MSO patients.

**Subjects**	**No. of patients**	**Mean ± SD**
Age (year)	143	42.55 ± 13.59
Age (live)	126	41.79 ± 12.85
Age (death)	6	56.83 ± 18.97
Tumor size (diameter/cm)	110	8.65 ± 4.38
Follow-up time (month)	75	64.77 ± 81.46

### Clinical Features

Some patients had a long history of thyroid disease. Among the 144 patients, 15 patients (11.11%) had thyroid nodules, 4 patients (2.78%) had hyperthyroidism, 8 patients (5.56%) had PTC, 2 patients had simple goiter (1.39%), and 1 patient had Hashimoto's thyroiditis (0.69%). In total, 114 patients (78.47%) had normal thyroids.

### Tumor Characteristics

The average diameter of the tumors in 110 samples with detailed description of the tumors was 8.65 ± 4.38 cm (Table 1), which is much larger than that of thyroid cancer (17). The tumors had significant propensity as follows: 51.88% involved the left ovaries, 39.85% involved the right ovaries, and 8.27% involved the bilateral ovaries.

### Diagnosis

Based on the included samples, the misdiagnosis rate of MSO was 17.27%. In our study, “impure” types accounted for 70.19% of the cases, and pure types accounted for 29.81% of the cases. In addition, our study included cases of mature cystic teratoma, even if the thyroid tissue composition was <50% of the lesion, but there was clear macroscopic invasion and metastasis of malignant thyroid tissue.

According to the diagnostic criteria, our study included the following 6 types of MSO: typical PTC, which accounted for 50.00% (51/102); follicular thyroid carcinoma, which accounted for 26.47% (27/102); follicular variant of papillary thyroid carcinoma (FVPTC), which accounted for 18.63% (19/102); papillary and follicular mixed thyroid carcinoma, which accounted for 2.94% (3/102); anaplastic carcinoma, which accounted for 0.98% (1/102); and medullary carcinoma, which accounted for 0.98% (1/102). In the diagnosed patients, 51.16% were positive for CA125 (>35 U/ml).

### Treatment

Overall, 58.57% of the patients underwent bilateral salpingo-oophorectomy, 35.71% of the patients underwent unilateral salpingo-oophorectomy, and 5.72% of the patients did not undergo salpingo-oophorectomy. In addition, 41.43% of the patients underwent total thyroidectomy, 32.14% of the patients received radioactive iodine after salpingo-oophorectomy and thyroidectomy, 2.86% of the patients underwent only thyroidectomy and radioactive iodine treatment, 1.43% of the patients underwent only simple tumor resection, 0.71% of the patients did not undergo surgical treatment, and 0.71% of the patients only received radioactive iodine treatment without salpingo-oophorectomy or thyroidectomy. Because MSO was rarely diagnosed before surgery, the patients were usually treated as suspicious ovarian tumors. Our research indicated that receiving radioactive iodine treatment was not significantly related to a death outcome (*P* > 0.05) (Table 2).

**Table 2:** Relationship between influencing factors and death.

**Influencing factors**		**Number of deaths**	**Total**	**Percentage**
	**Sort**			
Tumor	Yes	5	61	8.2%^*^
Metastasis	No	1	56	1.8%^*^
Radioiodine	Yes	2	52	3.8%
Therapy	No	4	75	5.3%
Pathological	FTC	0	31	0%^**^
Typing	PTC	2	58	3.4%^**^
	FVPTC	2	28	7.1%^**^
	Mixed	1	1	100%^**^
	AC	0	1	0%^**^

*FTC, follicular thyroid carcinoma; PTC, papillary thyroid carcinoma; FVPTC, follicular variants of papillary thyroid carcinoma; Mixed, papillary and follicular mixed thyroid carcinoma; AC, anaplastic carcinoma*.

* *P < 0.05*;

** *P < 0.01*.

### Prognosis

The statistical analysis of the samples with a clear follow-up time revealed that the average follow-up time was 64.77 ± 81.46 months, the longest follow-up time was 408 months, and the shortest follow-up time was 1 month (Table 1). The SEER database of the United States and cancer statistics of Japan show that the recent mortality rate from thyroid cancer in the United States was 0.5 per 100,000 men and women per year (18). Differing from thyroid cancer, the mortality rate of MSO was higher. Of the 127 patients with clear follow-up outcomes, 6 (4.72%) died (19–24). Most of the dead occurred in patients with distant metastasis (Table 2). The most common metastatic site was the lung. One patient died of respiratory failure due to lung metastasis (20). Another patient died of myeloma (23), which is not sure if it is caused by metastasis. Among the dead patients with a definite cancer type, 2 cases had papillary thyroid carcinoma, 2 had FVPTC, and one had mixed papillary and follicular carcinoma (Table 2) (*P* < 0.01); however, no patients with simple papillary thyroid cancer died. The mortality in the radioiodine treatment group was reduced by 1.5% compared with that in the patients who did not receive radioiodine treatment, but the difference was not statistically significant (*P* > 0.05) (Table 2).

### Molecular Biology

Ciccarelli believed that the tumor originates from a germ cell with several chromosomal abnormalities (25). Therefore, molecular biology marker screenings were conducted. The genes tested in our enrolled studies included *BRAF, NRAS, KRAS, HRAS, KIT, PIK3CA, RET, APC, DICER1, PRAKR1A, PTEN, TSHR, WRN, DDR2, EGFR, ERBB2, FGFR1, FGFR3, MET, PDGFRa*, and *TP53* (Table 3). Of the 6 patients undergoing genomic profiling with PTC and FVPTC, five patients were found to have mutations in one or more PTC-related disease genes, including the *BRAF, NRAS, KRAS*, and *KIT* genes (Table 3), while there were no genetic mutations detected in the patients with follicular thyroid carcinoma.

**Table 3:** Summary of gene mutations in MSO patients.

**Study**	**Cancer type**	**Gene mutations**	**Protein**
Coyne et al. (26)	FVPTC	NRAS codon 61C > A	gln → lys
Wolff et al. (27)	FVPTC	BRAF exon 601A > G	lys → glu K601E
Luo et al. (28)	PTC	KRAS codon12 G > T	gly → val
Seifert et al. (29)	FVPTC	None	None
Tsukada et al. (30)	FTC	None	None
Gild et al. (31)	FTC	None	None
Ma et al. (13)	FVPTC	KITc.1588G > Ac.1621A > C	p.V530I p.M541L
Flavin et al. (32)	PTC	BRAF codon 600T > A	T1799A val → glu

## Discussion

Abdominal symptoms were the main symptoms at the onset of MSO. Most patients complained of abdominal lumps. A few patients with metastases visited their doctors with the main complaints of tumor metastasis symptoms. MSO can also be accompanied by cancer-related obstructive symptoms, such as abdominal pain, bloating, and urinary incontinence. In a report by WOFF et al. (27), although 12% of the patients had acute abdominal pain (33), as many as 40% of the cases (5) were asymptomatic, which undoubtedly increases the probability of a delayed diagnosis. This disease often has gynecological symptoms, such as irregular vaginal bleeding and postmenopausal bleeding. Ascites has also been reported in 17% of the cases, but the ascites rarely contained malignant cells (34).

In our study, although 8 cases had thyroid carcinoma in thyroid glands, all authors noted that ovarian metastasis of primary thyroid carcinoma was extremely rare and the ovarian tumors were therefore thought to be independent primaries (35, 36). In some cases, the histological difference between thyroid cancer in the neck and ovarian thyroid cancer supports the independence of the two lesions (37). Siegel (16) noted that multifocal thyroid-type tumors arising synchronously in separate locations likely represent a common genetic predisposition for tumor formation early in its development. The initial genetic “hit” may occur during embryogenesis, resulting in cells with a common molecular sensitivity to tumor transformation (e.g., loss of genomic stability); these cells eventually differentiate into thyroid-type tissue and reside in two distinct anatomical sites during fetal development (38). After concurrent exposure to carcinogens subsequently in life, these thyroid cells undergo simultaneous but independent carcinogenesis and natural selection, resulting in lineage-specific carcinogenic expression (38).

Our research results showed the tumors had significant propensity in location distribution. This result differs from the research reported by Goffredo. His studies show that this tumor is equally distributed between the left and right ovaries (50% each) (3). We believe that the reason for this difference may be that his study included only 68 patients in the United States. In previous studies, the excess of right-sided ovarian germ cell cancers was evident in most age groups (39), and our result was the opposite. A larger sample size may be needed to explain this discrepancy. Studies have shown that tumors can spread to the contralateral ovary through blood-borne dissemination (21); thus, we speculate that due to the rarity of this disease, when patients are diagnosed, it is very likely that metastasis occurs before bilateral ovarian involvement occurs.

Malignant struma ovarii presents with the typical pathological features of PTC, including overlapping “ground glass” or “clear” nuclei, nuclear grooves, and sometimes papillary structures. Follicular thyroid carcinoma is not judged as benign or malignant by histomorphology. Similar to primary thyroid follicular carcinoma, the diagnosis of ovarian thyroid follicular carcinoma relies on invasion of the capsule or blood vessels, infiltration of neighboring organs and distant metastasis. The diagnostic criteria for follicular MSO are difficult to apply. For example, ovarian tumors usually do not have an envelope. The disappearance of the envelope is a basis for the diagnosis of follicular cancer. Invasive vesicle wall invasion is an important malignant feature, but in ovarian follicular carcinoma, there is often no cyst wall, which undoubtedly increases the difficulty of diagnosis. Therefore, infiltration of surrounding ovarian tissue, vascular invasion, and metastasis can be used as a basis for the diagnosis of malignancy (34). Bifulco et al. (40) thought that the assessment of Ki67 could be a valid support in the diagnosis of a more aggressive ovarian related tumor.

Follicular variants of papillary thyroid carcinoma (27) are diagnosed when the typical nuclear features of PTC are present without a papillary structure (37). Although some studies note that the criteria for disease diagnosis are controversial (32, 41), in the diagnosis, the criteria for thyroid cancer must be applied to the diagnosis of MSO (33).

Evidence of thyroid properties in ovarian tumors includes thyroglobulin, which can be used as a tumor marker of recurrence after thyroidectomy, I-131 ablation, and thyroid suppression (42, 43). Any increase in thyroglobulin should prompt follow-up whole body I-131 scans to search for relapse (41, 42, 44). DeSimone et al. (14) postulated that MSO is a tumor that secretes hormones autonomously and is stimulated by thyroid stimulating hormone receptor antibodies. Thyroglobulin is a glycosylated protein that provides a matrix for the synthesis and storage of thyroid hormones (45, 46). Thyroglobulin is a sensitive and valuable tumor marker that can be used for the clinical monitoring of struma ovarii and thyroid cancer (46, 47). If it is difficult to confirm benign or malignant disease, the diagnosis can be further confirmed by the detection of three proteins, including CK19 (48), galectin-3 (49), and CD56 (50, 51). The sensitivity of diagnosis based on the joint detection of these 3 proteins is 100% (52). In the diagnosis, it is very important to exclude the possibility of primary thyroid cancer metastasis to the ovary. If the ovarian cystic solid tumors and solid areas are rich in blood vessels on ultrasound (53), especially high-enhanced signals in ovarian polycystic tumors during CT or MRI examinations, more support for the diagnosis of struma ovarii is provided (54). Ultrasonography can be used as auxiliary methods for diagnosis (55).

Among the diagnosed patients, 51.16% were positive for CA125 (>35 U/ml). Regarding the low positive rate, one perspective argues that CA125 is not a specific tumor marker of MSO but may be a marker of ovarian germ cell tumors (41, 56). Another perspective posits that CA125 is related to age as the specificity of CA125 is lower before menopause. Among those aged <50 years who had a CA125 >35 U/ml, only 15% had ovarian cancer, and among those older than 50 years with an elevated CA125, up to 80% had cancer. CA125 has a sensitivity of 71–82% and a specificity of 75–94% in the diagnosis of malignant tumors (57). Compared with good specificity, the CA125 does not have an optimal sensitivity (58). The misdiagnosis rate of MSO was 17.27%, and most often, MSO was misdiagnosed as a benign ovarian teratoma or primary gynecological tumor (19). Pathological misdiagnosis accounts for the majority.

For PTC, medullary carcinoma and anaplastic carcinoma, traditional pathologic features of these tumors are usually enough for their diagnosis as primary thyroid tumors. It is only for well-differentiated follicular carcinoma that tissue morphology alone may not be sufficient, in this situation, obvious invasion or metastasis is necessary. The peritoneal spread of ruptured benign goiter is difficult to distinguish from well-differentiated carcinoma, highlighting the importance of referring the original surgical specimen of thyroid tissue to an experienced pathologist (59). Furthermore, the immunohistochemical and ultrasound examinations mentioned above are necessary.

Strumal carcinoid can be easily confused with thyroid papillary carcinoma. The most common MSO is papillary carcinoma. The ground glass nuclei, nuclear groove and nucleus of papillary carcinoma inclusion bodies have the most diagnostic value for thyroid papillary carcinoma. Furthermore, neuroendocrine markers of strumal carcinoid stain positive and both can be distinguished (60). Ovarian medullary thyroid carcinoma sometimes has carcinoid histological features; thus, strumal carcinoid can easily be confused with ovarian medullary thyroid carcinoma. Although some neuroendocrine markers of ovarian medullary thyroid carcinoma can also be positive, strumal carcinoid tumors rarely show positive calcitonin and rarely have amyloid deposits. The above characteristics might be helpful in distinguishing strumal carcinoid tumors from ovarian medullary thyroid carcinoma.

In serous cystadenocarcinoma, follicles with colloid do not appear, the luminal border of the epithelial cells stains positively with PAS and Alcian blue stains, neoplastic cells have decreased amounts of cell membrane interdigitations, mucus droplets may be present in the apical portions of many cells, no follicles are formed, and tumor cells lack oncocytic changes (61), they are distinguishable by light microscopy alone. In ambiguous cases, immunohistochemistry can be used. Specifically, primary ovarian serous carcinomas will be positive for PAX-8, WT-1, ER, and PR, and negative for thyroid markers such as TTF-1 and thyroglobulin (62). Differential diagnosis is not difficult according to the characteristics of thyroid cancer, but attention should be paid to differentiate it from cystadenocarcinoma.

In the differential diagnosis with polycystic ovary syndrome, it has a clear history of dysmenorrhea, generally with repeated abnormal bleeding. During MRI examinations, the T2WI signals are diverse, and low-signal cysts can be observed (63). T1WI is generally high-signal and can adhere to the surrounding tissue at a sharp angle, and the solid component of the enhanced scan is generally not significantly enhanced. Patients with tubal and ovarian abscesses often have symptoms, such as fever and abdominal pain, with elevated white blood cells and neutrophils. The cystic cavity is generally low-density or has long T1 and T2 signals, and DWI is a high signal (64), with obvious diffusion limited scanning that is enhanced similar to a honeycomb.

The standard treatment for patients with MSO is conservative surgery (total abdominal hysterectomy with bilateral salpingo-oophorectomy), thyroid hormone inhibiting therapy and complete surgical staging, including cytological abdominal washing, pelvic and para-aortic lymph node sampling, and omentectomy (3, 16, 56, 65). For young women who desire to retain fertility, unilateral oophorectomy or simple tumor resection is recommended (66). After successful fertility, regular follow-up should be performed. Nevertheless, total abdominal hysterectomy and bilateral salpingo-oophorectomy surgery are the primary choices (67).

Unfortunately, our research indicated that receiving radioactive iodine treatment was not significantly related to a death outcome. As with any such review, the study is limited by the quality and detail provided in the previously reported cases. Nonetheless, risk stratification might be helpful. Yassa et al. (68) proposed that patients with tumors >2 cm, extra-ovarian disease or aggressive histological features should consider thyroidectomy and eventually undergo I-131 ablation. Janszen et al. (69) suggested that MSO with a diameter ≥1 cm should be treated with I-131 ablation after thyroidectomy. On the one hand, thyroglobulin is mainly secreted by the thyroid gland; thus, thyroglobulin can be regarded as a tumor marker of relapse only when the thyroid glands of the patients have been completely removed (70). On the other hand, thyroglobulin can rule out the possibility of cervical thyroid cancer metastasis.

This report is the first in the literature to conclude that the outcome is related to the type of cancer. Studies have increasingly advocated for radioactive iodine therapy (12), but our study found that the mortality in the radioiodine treatment group was reduced by 1.5% compared with that in the patients who did not receive radioiodine treatment, but the difference was not statistically significant. As mentioned above, MSO is rare and difficult to find and diagnose, especially in those with non-papillary thyroid cancer. Similar to thyroid cancer in the neck, MSO is a very mild cancer with low lethality. It is difficult to analyse the risk factors of mortality and the efficacy of therapeutic options without a large enough sample size. Nevertheless, age, the type of cancer, and metastasis might be related to the mortality of MSO.

*BRAF* mutations have been confirmed in a case report of ectopic thyroid cancer (29). Xing et al. (71) showed that *BRAF* mutations can independently predict advanced disease in thyroid cancer located in the thyroid gland. However, regarding MSO, more research and longer follow-up are needed before conclusions. In addition, RAS mutations were related to FVPTC (72), and regarding this finding, a consensus was reached in the pathology community (73–75).

In one case (13) of a *KIT* mutation, because of the coexistence of PTC in the neck, interference from the neck could not be ruled out. *KIT* gene mutations have been reported in invasive fibroids, acute myeloid leukemia, and colon adenocarcinoma (76), but the p.V530I mutation was reported for the first time. Because knowledge regarding this rare mutation is limited, its significance is currently unclear; however, the p.V530I mutation has not been reported in thyroid cancer in the neck.

## Summary

MSO is a rare malignant tumor of the reproductive system, which has various biological invasive behaviors that are similar to those of thyroid cancer. Thyroid carcinoma arising in struma ovarii should prompt clinical evaluation and imaging of the thyroid taking into account the possibility of primaries, metastases and recurrence. The prognosis is closely related to the age, type of cancer and distal metastasis of the patient. Surgery, radioactive iodine treatment and TSH inhibiting therapy were the most commonly used treatment options in our enrolled studies.

## Author Contributions

YC: Document Retrieval, Data Extraction, Data analysis, Essay writing, and Paper submission. JY, SW, and JZ: Data Extraction and Data analysis. JD: Article innovation. LL: Article innovation and Paper submission. All authors contributed to the article and approved the submitted version.

## Conflict of Interest

The authors declare that the research was conducted in the absence of any commercial or financial relationships that could be construed as a potential conflict of interest.

## Abbreviations

MOS: malignant struma ovarii
PTC: papillary thyroid carcinoma
FTC: follicular thyroid carcinoma
FVPTC: follicular variant of papillary thyroid carcinoma
gln: glutamine
lys: lysine
glu: glutamic acid
gly: glycine
val: valine.
